# Surgical Management of Stage IV Melanoma: Clinical, Molecular, and Therapeutic Considerations

**DOI:** 10.3390/ijms27052327

**Published:** 2026-03-02

**Authors:** Ifeanyi K. Uche, John M. Lyons

**Affiliations:** 1 Department of Surgery, Louisiana State University Health Sciences Center, New Orleans, LA 70112, USA; iuche@lsuhsc.edu; 2 Our Lady of the Lake Cancer Center, Baton Rouge, LA 70808, USA

**Keywords:** stage IV melanoma, metastatic melanoma, surgery, oligoprogression, gene profiling, circulating tumor DNA, molecular drivers, systemic therapy

## Abstract

Despite major advances in systemic therapy, surgery still plays a valuable role in the management of metastatic melanoma. This review summarizes the historical evolution of surgical management, outlines the traditional clinical risk factors, and examines biochemical, molecular, and mutational factors that impact melanoma tumor biology. Emerging tools such as predictive biomarker assays, gene expression profiling, and circulating tumor DNA are discussed in the context of patient selection. Finally, we consider contemporary indications for surgery, the management of oligoprogression, sequence of treatment, and optimal timing of resection while highlighting how operative intervention integrates with modern melanoma care.

## 1. Introduction

Cutaneous melanoma is an aggressive malignancy that affects over 200,000 people in the United States annually [1]. The 5-year survival rate for patients diagnosed and treated at an early stage approaches 99%. However, survival decreases significantly with more advanced stage—approximately 75% 5-year survival for patients with regional spread and less than 30% for those with distant metastatic disease [1].

The advent of immune checkpoint inhibitors (ICIs), particularly the combination of anti-cytotoxic T lymphocyte-associated antigen-4 (CTLA-4) and anti-programmed death receptor-1 (PD-1) agents, has dramatically transformed the prognosis of metastatic melanoma. Recent long-term data from seminal trials such as CheckMate 067 have shown overall survival exceeding 10 years (120 months) in a subset of patients receiving dual-ICI therapy, far surpassing outcomes traditionally observed with surgery alone. Despite this, surgery can still play a valuable role as a complementary treatment in select patients.

This review examines surgical management of metastatic melanoma, key clinical and molecular factors, emerging predictive tools such as biomarker assays, gene expression profiling (GEP), and circulating tumor DNA (ctDNA), as well as contemporary indications for surgery in the era of modern systemic therapy.

## 2. Historical Perspective of Surgery in Metastatic Melanoma

The first widely accepted surgical excision of what was later identified as metastatic melanoma was performed by the Scottish surgeon, John Hunter, in 1787 [2]. He removed a “cancerous fungous excrescence” from behind the angle of the jaw of a 35-year-old man [2]. The removed tumor was preserved, and in 1968, microscopic re-examination of the specimen revealed it to be metastatic melanoma [3].

The first modern report of metastatectomy was a documented pulmonary resection of metastatic melanoma by Arce in 1936 [4]. Three years later, Ochsner and DeBakey reported a pneumonectomy for melanoma within a series of 79 pneumonectomies conducted for various malignancies [5]. In 1942, Carlucci and Schleussner described performing simple pneumonectomies specifically for melanoma [6]. In 1948, a case report in the Case Records of the Massachusetts General Hospital detailed a pulmonary resection of metastatic melanoma in a 70-year-old retired merchant [7].

These initial studies were descriptive, but they lacked comprehensive detail on outcomes. In 1973, Cahan reported a series of 29 (1%) patients who underwent resection for metastatic melanoma to the lung from a database of 2500 patients seen from 1949 to 1970 at the Memorial Sloan Kettering Cancer Center. He reported a 5-year survival of 14% for the entire surgical cohort [8]. Acknowledging the limitations of this small series, Cahan considered it “not unreasonable to suppose that the removal of as much tumor as possible wherever and whenever it is feasible” is more preferred than leaving it in place. These results were subsequently challenged when Mathisen et al., in 1979, reported no 5-year survivals among resected patients with pulmonary metastasis from melanoma, and no difference in overall survival (OS) in patients who underwent surgery versus those who did not [9]. Similar results were reported by Dahlback et al., who also observed no long-term survivals, leading them to conclude that lung surgery did not contribute to the survival of patients with pulmonary metastasis [10].

Following these early historical reports, the role of surgery in metastatic melanoma remained controversial and limited. In the early 1990s, however, Harpole et al. reported on a large surgical series of patients having metastasectomy from a database of 1000 patients with pulmonary metastasis. They found that complete surgical resection was independently associated with improved OS, with a 5-year survival rate of 20% [11]. Shortly thereafter, Tafra et al., at the John Wayne Cancer Institute, analyzed over 980 patients with pulmonary metastases, including 106 who underwent surgical resection. Surgical patients had markedly improved OS, with a 5-year survival of 27% versus 3% in non-surgical patients, and multivariate analysis again identified complete resection as an independent predictor of survival [12].

These seminal reports initiated an era in which resection of metastatic lesions became an accepted strategy for appropriately selected patients with stage IV melanoma. Subsequent series have reinforced these findings, consistently demonstrating that patients selected for metastasectomy can experience legitimate improvements in OS.

## 3. Prognostic Factors in Metastatic Melanoma to Consider in the Surgical Patient

### 3.1. Clinical Factors

In general, patients with more indolent, smaller-volume metastasis have better outcomes with surgery than those with rapidly progressive disease, illustrating the importance of tumor biology and patient selection [13]. Over time, several authors have identified clinical factors that are predictive of survival following complete metastatic resection. These include the number of metastatic lesions, the number of metastatic sites, the disease-free interval (DFI) prior to the development of metastasis, tumor doubling time (TDT), thickness of the primary, and the anatomic location of metastasis.

The number of metastases is a well-recognized prognostic factor for OS in metastatic melanoma [14,15,16]. A large study providing evidence for this analyzed 1574 patients who underwent surgery for metastatic melanoma. The authors found a 5-year survival rate of 29% in patients with a single metastatic deposit, compared to just 11% in those with four or more metastases [17]. The significance of the number of metastatic sites was highlighted in patients monitored following the Multicenter Selective Lymphadenectomy Trial (MSLT) I. Howard et al. reported on 291 trial patients with stage IV melanoma recurrence. They demonstrated that OS decreased for patients undergoing metastatectomy as the number of metastatic sites increased. Patients who had disease resected from one metastatic site had a median survival of 17 months compared with 13 and 4.5 months for patients with two and three or more involved organs, respectively [15]. It is notable that a phase III randomized trial designed to evaluate an allogeneic whole-cell vaccine following resection of metastatic melanoma allowed enrollment of patients with up to five metastatic deposits. However, fewer than 5% of trial participants enrolled actually had more than 3 metastatic lesions [18]. These studies also noted improved OS in association with longer disease-free interval. Patients with at least a 36-month DFI experienced a median OS of 30 months, while those with a with DFI less than 12 months experienced a median OS of only 9.3 months [15,17].

The rate at which a tumor increases in size, assessed by TDT, is calculated by measuring the changing diameters of each tumor nodule. TDT has been correlated with patient survival across multiple malignancies since the 1950s [19,20]. In a study from the John Wayne Cancer Institute, a TDT greater than 60 days in melanoma patients with pulmonary metastases was associated with a 5-year OS rate of 20.7%, compared to a 5-year OS rate of 0% when the TDT was less than 60 days [21].

Thickness of the primary melanoma has also been shown to be an important predictor of survival in patients with metastatic disease [7]. In a cohort of 227 Australian patients with stage IV melanoma, Luen et al. reported that primary tumor thickness was an independent predictor of OS. They found that for every additional millimeter of tumor thickness, the hazard of death in the metastatic patient increased by 9% [22]. This relationship between tumor thickness and prognosis in metastatic disease has also been observed in several other retrospective and prospective studies [18].

The location of metastatic lesions is a powerful predictor of survival in patients with melanoma, so much so that it is reflected in the American Joint Committee on Cancer (AJCC) staging system. Stage IV melanoma that is M1a denotes metastases to skin, subcutaneous tissue, muscle, or distant lymph nodes, and it is associated with the most favorable prognosis. M1b disease includes pulmonary metastases (with or without concurrent M1a sites). M1c encompasses metastases to other visceral organs, excluding the central nervous system (CNS), and M1d denotes CNS involvement, which carries the worst prognosis. Clinical outcomes correlate well with this stage classification. In the CheckMate 067 trial, patients with M1a or M1b disease treated with dual-ICI achieved a 10-year melanoma-specific survival (MSS) of 52%, compared to only 43% MSS for patients with M1c disease [23]. Despite the substantial survival improvements achieved with modern systemic therapies across all metastatic sites, a survival gradient based on the location of metastases persists to this day; this should be considered when assessing treatment options.

### 3.2. Biochemical Factors

Lactate dehydrogenase (LDH) is a prognostic serologic biomarker that has been incorporated into the AJCC staging system for stage IV melanoma since the sixth edition [22,24]. Elevated LDH is consistently associated with worse OS, including among patients undergoing surgical resection of metastatic disease. In a retrospective study of over 200 patients with resected metastatic melanoma, normal pre-operative LDH correlated with a median OS of 13 months, compared with only 6 months for patients with elevated LDH. The outcomes were considerably worse when the LDH exceeded two-fold above the upper limit of normal [25]. While LDH demonstrates high specificity as a tumor marker, its relatively low sensitivity limits its utility as a standalone prognostic indicator [26,27].

S100B, a calcium-binding protein expressed in melanocytes, provides complementary prognostic information, and it may be more melanoma-specific than LDH. Retrospective analyses have shown that elevated S100B predicts poorer survival in advanced melanoma, independent of LDH [28,29]. In contemporary patients treated with ICIs, rising S100B levels during therapy correlated strongly with worse OS, whereas changes in LDH were less consistently predictive [30]. In the surgical setting, S100B has also demonstrated utility. Among patients undergoing complete metastasectomy, patients with a normal pre-operative S100B experienced improved OS compared to those with elevated levels; rising post-operative S100B often preceded clinical or radiologic recurrence [25,30]. Similar to the limitations seen in LDH, the suboptimal sensitivity and specificity of S100B also constrain its efficacy as a standalone decision tool. However, a 2021 meta-analysis directly comparing S100B and LDH concluded that while neither marker is definitively superior, their combined usage is better to inform patient selection than either marker is individually [28].

Several additional serum and molecular biomarkers have also been investigated in melanoma, including tyrosinase, melanoma-inhibitory activity (MIA), MART-1/Melan-A, and alkaline phosphatase (AP). To date, however, LDH and S100B remain the only widely accepted serologic markers for prognostication in advanced melanoma.

### 3.3. Molecular and Mutational Factors

Molecular profiling provides insight into the distinct clinical patterns observed among different melanoma subtypes, and this can be used to guide treatment decisions in metastatic melanoma. Most cutaneous melanomas harbor ultraviolet (UV)-associated mutations affecting the MAP kinase (MAPK) pathway. Alterations in BRAF V600 are the most common event, occurring in approximately 50% of patients. BRAF V600 encodes a serine/threonine kinase that constitutively activates the MAPK/ERK signaling pathway, thereby promoting cellular proliferation and inhibiting apoptosis [31]. The majority of these mutations (~85%) involve a substitution of glutamic acid for valine at codon 600 (V600E), which is particularly significant not only for its role in driving tumor growth, but also because it confers susceptibility to targeted BRAF and MEK inhibition.

Historically, BRAF mutants experienced worse OS. In a pooled meta-analysis of 52 studies comprising 7519 patients with malignant melanoma, the presence of a BRAF mutation was associated with worse OS, conferring a 23% increased risk of death across the combined cohorts [32]. In a retrospective cohort of 677 stage IV melanoma patients at M.D. Anderson Cancer Center, BRAF-mutant patients incurred a higher incidence of CNS metastasis at diagnosis, indicating BRAF’s association with more aggressive biology [14]. In the era of modern therapy, however, outcomes for BRAF-mutant metastatic melanoma have substantially improved. In a phase III randomized study of 423 patients with unresectable or advanced BRAF V600-mutant melanoma, combination BRAF/MEK inhibition achieved an overall response rate of 67% [33]. In a subsequent study, BRAF V600-mutant advanced melanoma patients treated with combined BRAF and MEK inhibition experienced a landmark 5-year OS rate of 34% [34]. Tumor response to these agents can be rapid and profound, making them especially valuable in patients with high disease burden, symptomatic metastases, or rapidly progressive disease.

BRAF V600-mutant tumors also respond to ICI. In treatment-naive individuals receiving first-line dual-ICI, 5-year OS rates have reached 60% [34]. In addition, the first-line ICI is associated with a survival rate above that seen with first-line targeted therapy [35]. In the IMMUNED phase II trial, in which adjuvant combination anti-PD-1 and anti-CTLA-4 was given following completely resected stage IV melanoma, recurrences among BRAF-mutant patients were observed in only 19%, compared to 45% seen in BRAF wild-type patients [36]. However, in the PRADO study, which offered limited nodal surgery to stage III patients who achieved a major pathologic response (MPR) following neoadjuvant ICI therapy, recurrences were predominantly observed in patients with BRAF mutations [37]. These findings underscore the challenges and complexities in predicting treatment outcomes based on BRAF mutation status, and highlights the need for further research in this area. Nevertheless, patients who achieve a durable response to targeted or ICI therapy may be excellent candidates for consolidative metastatectomy in cases where small-volume disease remains. However, patients with BRAF-mutant disease who progress early on targeted therapy harbor a more aggressive biology, and surgery is less likely to offer a durable benefit. Thus, resection should be considered very cautiously in these individuals.

NRAS was the first oncogene to be described in melanoma in 1984 [38]. Clinically, NRAS-mutant melanomas exhibit more aggressive features—including greater tumor thickness, higher mitotic rates, and increased lymph-node involvement—contributing to worse OS compared with NRAS-wild-type disease [39]. Although NRAS mutations occur in approximately 15–20% of cutaneous melanomas, there are no approved therapies that target this mutation at this time. MEK inhibitors have shown activity in NRAS-mutant melanoma, with binimetinib demonstrating improved response rates and progression-free survival (PFS) compared with dacarbazine. However, the observed responses are modest [40].

Retrospective analyses evaluating surgical outcomes in NRAS-mutant melanoma remain limited, but two studies provide important insights. In a series of patients undergoing pulmonary metastasectomy, 20% harbored NRAS mutations; these patients demonstrated a trend toward shorter RFS and OS following surgery. Additionally, multivariable modeling identified the presence of an NRAS mutation as an adverse prognostic factor [41]. In another retrospective series of 159 patients who underwent sentinel lymph node (SLN) biopsy, NRAS mutations correlated with significantly worse OS compared with BRAF-mutant or wild-type melanoma despite no association with SLN positivity [42]. These findings imply that NRAS mutations confer a higher-risk phenotype not fully captured by nodal staging.

Because seminal ICI trials did not report NRAS-specific outcomes, evidence for their efficacy in NRAS-mutant melanoma is primarily derived from retrospective series. These studies show that NRAS-mutant tumors respond to ICI with rates comparable to or slightly higher than those seen in BRAF-mutant or NRAS-wild-type melanoma [43,44,45]. These findings are consistent with at least three more recent studies conducted by members of the International Neoadjuvant Melanoma Consortium (INMC), in which NRAS-mutant melanoma patients achieve similar major pathological response (MPR) rates to wild-type melanoma patients when treated with neoadjuvants ipilimumab and nivolumab [37,46,47]. These findings suggest that neoadjuvant ICI remains highly effective regardless of NRAS mutation status, potentially neutralizing the virulence traditionally observed in NRAS-mutant tumors. Moreover, MPR was the primary and most significant predictor of long-term survival. This implies that, in the era of neoadjuvant ICI, NRAS status is a less significant biomarker than MPR in determining the efficacy of surgery in stage IV melanoma.

NF1 mutations occur in approximately 10–15% of melanomas, and they are characterized by high tumor mutational burden (TMB) and marked genomic heterogeneity—features that may enhance tumor immunogenicity. Preclinical data imply that loss of NF1 is associated with increased PD-L1 expression, an effect reversible with ICI therapy [48]. Retrospective analyses indicate that NF1-mutant melanoma can respond to ICI, and some cohorts have reported improved OS relative to NF1-wild-type disease [49]. However, results have been inconsistent [50]. Despite growing interest in the implications of NF1 alterations, data on surgical outcomes in this molecular subgroup remain sparse. Although a potentially relevant molecular marker, current evidence is insufficient to guide surgical decision-making based on NF1 mutation status, and further research is needed to clarify its role in treatment selection [51].

The presence of a nodular subtype has been shown to impact the outcome of patients with metastatic disease. Investigators from New York University used both an institutional dataset and the SEER database to compare outcomes between nodular and superficial spreading melanomas in both primary and metastatic disease [52]. After controlling for thickness, ulceration, mitotic rate, and AJCC stage, nodular patients had a higher risk of death in both databases, implying an underlying mutational difference between these subtypes. This was confirmed when the authors noted a higher frequency of NRAS mutations and a lower mutational rate among eight different somatic driver genes within nodular subtypes. While nodular patients experienced a lower response rate to BRAF-targeted therapy, no response differences or survival differences were observed among patients receiving ICI based on tumor subtype [52]. This was also illustrated in a large population-based analysis from the Dutch Melanoma Treatment Registry [53]. Both studies found no significant difference in survival following ICI between patients based on nodular or superficial spreading subtype. Thus, the adverse prognosis traditionally linked to the nodular subtype may be mitigated in the modern immunotherapy era.

Acral melanomas (AMs) represent another biologically distinct subtype with worse outcomes. Arising on the palms, soles, and nail beds, they develop independent of UV exposure. Accordingly, they harbor fewer UV-induced mutations and generally exhibit a lower TMB than non-acral cutaneous melanoma. The TMB in AM has been reported as roughly 1.5 mutations/mB versus 9.5 mutations/mB generally reported in other subtypes of cutaneous melanoma [54]. This may account for the pattern of response to ICI observed in AM. In a multicenter cohort of 325 patients with unresectable stage III–IV AM, the first-line objective response rate (ORR) to treatment with ICI combination therapy was 43%. However, this ORR was not translated into improvement in either PFS or OS [55]. While BRAF and NRAS mutations are less frequently observed in AM, c-KIT alterations occur in a substantial number of AM patients, and this provides a strong rationale for treatment with KIT inhibitors such as imatinib [56]. However, the clinical data for AM patients treated with KIT-directed therapy remain limited and mixed. In a phase II trial of imatinib in metastatic melanomas with KIT amplifications or mutations (including AM), the best overall response rate was 29% [56]. This was also observed in a more recent pooled analysis of 130 KIT-altered melanoma patients in which AM patients experienced an ORR of 25%. Responding patients—especially those with KIT mutations in exons 11 or 13—demonstrated longer PFS and OS than those with less-responsive subtypes [57]. These observations confirm that while KIT alterations may provide a therapeutic opportunity in AM patients, only a subset of patients derive durable benefit. The lower response rates to systemic therapies contribute to their worse prognosis, and underscore the need to consider surgery very selectively in these individuals.

### 3.4. Additional Prognostic Tools

Programmed death receptor ligand-1 (PD-L1) is a transmembrane protein expressed on tumor and tumor-infiltrating immune cells that inhibits T-cell activity via binding to PD-1. It is assessed by immunohistochemistry, and it has emerged as an important biomarker in several malignancies—most notably non-small cell lung cancer, urothelial carcinoma, and head and neck squamous cell carcinoma. Multiple studies have evaluated whether PD L1 expression predicts response to ICI therapy, but, in melanoma, the predictive value is not clear. In the phase Ib KEYNOTE-001 trial, membranous PD-L1 expression was scored on a unique melanoma (MEL) scale of 0 to 5, where a score ≥ 2 was considered positive. The authors found that a higher PD L1 “MEL” score correlated with improved ORR, PFS, and OS. However, approximately 10% of patients who were PD-L1-negative still achieved response to ICI therapy (MEL 0 ORR = 8%; MEL 1 ORR = 12%) [58].

A more recent meta-analysis, pooling data from 13 studies and over 1000 patients, observed that high PD-L1 expression did not correlate with PFS and OS, but was instead linked to fewer lymph node metastases. These authors concluded that, although PD-L1 staining indicates different disease characteristics, it is not a reliable prognostic marker for survival [59]. In the OpACIN-neo trial, authors studied different regimens of ipilimumab and nivolumab prior to resection of macroscopic stage III melanoma [46]. They found that 60% of patients had an MPR, and that this was the strongest predictor of disease recurrence. However, when comparing patients with <1%, 1–50%, and >50% PD-L1 expression, no correlation was found between PD-L1 expression and recurrence rates. This further highlights the limited role of PD-L1 in predicting outcomes in melanoma. Thus, this test should not be used to inform surgical treatment at this time [60].

Circulating tumor DNA (ctDNA) consists of small fragments of tumor-derived DNA that shed into the bloodstream, which are subsequently captured for analysis. This tool is a robust, minimally invasive biomarker that has several potential applications in melanoma, including prognostication, disease monitoring, and assessment of treatment response (Table 1) [61,62,63,64,65]. Detectable ctDNA at the time of treatment has been consistently associated with worse outcomes independent of tumor stage. In a cohort of 161 high-risk stage II/III patients, the presence of BRAF or NRAS ctDNA in plasma corresponded to a 5-year survival of 33%, compared with 65% for patients with undetectable ctDNA [62]. These findings were further corroborated by a meta-analysis of over 600 patients across nine studies, as well as a systematic review of 26 studies primarily involving stage III/IV melanoma. Both of these large reviews confirmed that ctDNA detectability predicts worse OS [66,67]. Additionally, baseline plasma ctDNA has been shown to be a stronger independent predictor of disease progression than conventional serum markers such as S100B or LDH [68].

Beyond its baseline prognostic value, numerous studies have shown that rising ctDNA frequently signals impending relapse, and this has been a significant finding in surgical patients [68]. In a prospective study of 47 patients undergoing surgical resection of liver metastases from uveal melanoma, detectable ctDNA prior to surgery was associated with significantly shorter relapse-free survival (median RFS 5.5 vs. 12.2 months) compared to those with undetectable ctDNA. Post-operative ctDNA detection was also correlated with worse OS [69].

Taken together, the studies summarized in Table 1 collectively demonstrate the robust prognostic and predictive value of ctDNA in the management of advanced-stage melanoma. In the pre-treatment setting, detectable baseline ctDNA has consistently been associated with worse clinical outcomes and reduced response rates to systemic therapy. For instance, Seremet et al. reported that baseline ctDNA levels predicted response to pembrolizumab, and that decreasing ctDNA levels during follow-up were strongly correlated with improved OS [65]. Notably, patients who achieved ctDNA clearance had a significantly lower risk of death (HR 0.16, 95% CI 0.07–0.36; *p* < 0.001) [65]. Similarly, Bratman et al. demonstrated that ctDNA clearance during pembrolizumab therapy was associated with durable objective responses and a 100% overall survival rate at a median follow-up of 25.4 months (range 10.8–29.5) [70]. Furthermore, early on-treatment ctDNA assessment at week 2 or week 4 in patients receiving anti-PD-1 monotherapy or combination with anti-CTLA-4 therapy has shown promise in evaluating early biological response and identifying primary resistance to immunotherapy [71].

To date, prospective studies in metastatic cutaneous melanoma using ctDNA to guide surgical decisions are lacking. Nevertheless, emerging evidence suggests a potential role for ctDNA as an adjunctive risk-stratification tool in the consideration of metastasectomy. In a pilot prospective study of patients with stage IIB/C and resectable stage III melanoma, Brunsgaard et al. reported that 96% of patients, including those with detectable pre-operative ctDNA, had undetectable ctDNA levels within four weeks following surgery [72]. However, during post-operative surveillance, ctDNA levels were later detected in 6 of 20 patients, all of whom subsequently developed clinical recurrence [72]. Additional concerns also exist, such as the lack of assay standardization, variability in methodology (tumor-informed versus tumor-agnostic approaches), and differences in sensitivity thresholds. These further limit the standard adoption of ctDNA measurements in informing patients for surgical selection at this time [73,74].

Gene expression profiling (GEP) is a molecular diagnostic technique that evaluates the activity of multiple genes within a tumor to provide information about its biological behavior. One 31-gene expression profile (31 GEP) that is commonly used and commercially available classifies primary cutaneous melanomas into recurrence risk categories—Class 1A (low risk), Class 1B/2A (intermediate), and Class 2B (high risk)—based on tumor RNA expression [75]. Multicenter studies assessing this 31-GEP have demonstrated robust risk stratification. In a cohort of 523 patients with early-stage melanoma, 5-year RFS and DMFS was 88% and 93% for Class 1 patients compared to 52% and 62% for Class 2 patients, respectively (*p* < 0.001). The 31-GEP remained independently prognostic after adjusting for Breslow thickness, ulceration, mitotic rate, and sentinel lymph node status [76]. This was subsequently corroborated in a prospective analysis in stage IB–II disease (*n* = 86), where Class 2B tumors predicted a lower 3-year DFS (75% vs. 100%, HR 8.4, *p* = 0.008) [77]. Furthermore, both population-level and meta-analytic studies (*n* > 4500) have confirmed consistent prediction of MSS and OS, highlighting the test’s reproducibility and independent prognostic significance [77,78]. The predictive utility of GEP and other markers discussed have been summarized in Table 2.

Despite the evidence supporting its prognostic value in early-stage melanoma [79], GEP has limited data in patients with regional or distant metastases, and there exist no prospective studies demonstrating that GEP-guided management improves survival in metastatic melanoma. Additionally, GEP is currently not endorsed by national melanoma guidelines, and it is not recommended to replace standard clinical variables for guiding therapy or surveillance in early or advanced melanoma [80].

There are numerous additional tools, such as granzyme-B positron emission tomography (PET) imaging and gut microbiome analysis, which hold great promise for more precise prognostication [81]. However, their integration into routine clinical practice remains experimental currently.

**Table 1:** Overview of recent studies utilizing ctDNA in patients with melanoma.

Authors	Number and Population of Patients with Melanoma and Number	Setting	ctDNA Platform	ctDNA Time Point and Metrics	OS	Outcome
Syeda et al. [61]	870 patients with resected BRAF^V600^-mutant stage III melanoma	Post-treatment surveillance	BRAF mutation ddPCR	Detectable vs. undetectable	Placebo: 33.90 months [13.96-NR] vs. NR; HR 3.35 [2.01–5.55], combination therapy: 40.31 months [24.90-NR] vs. NR; HR 4.27 [2.50–7.27]	Placebo: RFS 3.71 months [95% CI 29.39–6.89] vs. 24.41 months [17.28–43.13], HR 2.91 [95% CI 1.99–4.25]; combination therapy: RFS 16.59 months [95% CI 12.02–26.80] vs. 68.11 months [50.36.28-NR], HR 2.98 [95% CI 1.95–4.54]
Lee et al. [62]	161 patients with stage II/III melanoma	Post-op	Mutation-specific ddPCR	Detectable vs. undetectable at baseline and post-op	Detectable ctDNA: 5 years OS = 33% [95% CI 14–55%]; undetectable ctDNA: 5 years OS = 65% (95% CI 56–72%); detectable ctDNA: median OS = 2.9 years [95% CI 0.9-NR] vs. NR [95% CI 6.0-NR] (undetectable)	Detectable: median DFI 0.3 years [95% CI 0.1–1.0] vs. 4.2 years [95% CI 2.5-NR] (undetectable); detectable: DMFI 0.6 years [95% CI 0.2–2.8] vs. NR [95% CI 5.0-NR] (undetectable)
Tan et al. [82]	133 patients with stage III resected cutaneous melanoma	Pre-op and post-op	NGS assay and ddPCR	Detectable vs. undetectable at baseline and post-op	Not reported	Baseline: RFS HR 2.9 [95% CI 1.5–5.6]; post-operative: HR 10 [95% CI 4.3–24; baseline DMFS HR 2.9 [95% CI 1.3–5.7]; post-operative: DMFS HR 10 [95% CI 4.3–27]
Lee et al. [83]	119 patients with stage IIIB/C/D melanoma	Pre-op	ddPCR	Detectable vs. undetectable at pre-op	Not reported	Detectable ctDNA median MSS: 17.6 months vs. 49.4 months (undetectable ctDNA) [HR 2.11 (95% CI 1.20–3.71)]; detectable ctDNA median DM-RFS: 6.2 months vs. 13.9 months (undetectable ctDNA) [HR 1.59 (95% CI 1.0–2.52)]
Seremet et al. [65]	85 patients with BRAF- or NRAS-mutant stage IV melanoma	Post-treatment surveillance	ddPCR	Detectable vs. undetectable at baseline and post-treatment	Undetectable baseline: median OS = NR vs. detectable baseline, OS 21 weeks (95% CI 0–43); undetectable baseline: 1 year SR = 70% (95% CI 54.3–85.7); 2.5 years SR = 54% (95% CI 34.4–73.6); detectable baseline: 1 year SR = 32% (95% CI 14.36–49.64); 2.5 years SR = 16% (95% CI 7.52–39.52)	Undetectable baseline: median PFS = 26 weeks (95% CI 0–71.1%) vs. detectable baseline, PFS 9 weeks (95% CI 6.9–11)
Herbreteau et al. [63]	53 patients with stage IV or non-resectable BRAF- or NRAS-mutant stage IIIc melanoma	Pre- and post-treatment surveillance	Mutation-specific dPCR	Detectable vs. undetectable at baseline and post-treatment	Undetectability at baseline: > 6 months survival rate (90.0% vs. 56.2% in detectable ctDNA)	Absent bR: 0% RR, 0% PFS rate at 120 days, median OS = 130 days; initial bR + bP: 0% RR, median PFS = 115 days, median OS = 148 days; persistent bR: 100% RR, and 100% PFS and OS rates for follow-up periods of 305 to 755 days
Herbreteau et al. [64]	49 patients with stage IV or non-resectable BRAF- or NRAS-mutant stage III metastatic melanoma	Post-treatment surveillance	Mutation-specific dPCR	Detectable vs. undetectable at baseline and post-treatment	Low/undetectable: 1 year OS = 81%; absent bP: 1 year OS = 13%; absent initial bP: 1 year OS = 73%; bP: 1 year OS = 81%	Low/undetectable: 4 months PFS = 80%; absent initial bP: 4 months PFS = 78%; absent bP: 4 months PFS = 78%; bP: 4 months PFS = 0%
Lee et al. [71]	86 patients with BRAF-, NRAS-, KIT-mutant stage IV metastatic melanoma	During treatment and post-treatment	Mutation-specific dPCR	Detectable vs. undetectable at baseline, during and post-treatment	Median OS (group A and B) = NR, 9.2 months for group C [HR 0.02]; total cohort: 1 year OS = 77%	RECIST response: group A 72%, group B 77%, group C 6%; median PFS: NR (group A [HR 0.08 (95% CI 0.03–0.20]) and B [HR 0.15 (95% CI 0.03–0.18]), 2.7 months (group C [HR 0.08 (95% CI 0.03–0.20]); total cohort: RR = 53%; 1 year PFS = 51%

Abbreviations: bR, biological response; bP, biological progression; CI, confidence interval; ctDNA, circulating tumor DNA; ddPCR, droplet digital polymerase chain reaction; DFI, disease-free interval; DMFI, distant metastasis-free interval; DMFS, distant metastasis-free survival; DM-RFS, distant metastasis recurrence-free survival; HR, hazard ratio; NR, not reached; NGS, next-generation sequencing; MSS, melanoma-specific survival; OS, overall survival; PFS, progression-free survival; RR, response rate; SR, survival rate. bR = statistically significant decrease in ctDNA concentration compared to baseline; bP = statistically significant increase in ctDNA concentration compared to nadir; group A = undetectable ctDNA at baseline; group B = elevated ctDNA at baseline but undetectable within 12 weeks of therapy; group C = elevated ctDNA at baseline and remained elevated during treatment.

**Table 2:** Prognostic and predictive clinical relevance of select biomarkers in metastatic melanoma.

Variable	Prognostic	Predictive
LDH	Yes—High LDH levels are associated with worse OS [25].	Yes—Early decrease in LDH during treatment corresponds to better response to therapy [84].
S100B	Yes—High S100B levels are associated with worse OS [28,29,30].	Yes—Early decrease in S100B during treatment corresponds to better response to therapy [84]. Patients with normal S100B prior to metastatectomy have improved OS following surgery [25,30].
BRAF V600E Mutation	Yes—BRAF-mutated tumors are associated with worse OS [14,32].	Yes—BRAF-mutated tumors demonstrate improved response to BRAF/MEK-targeted treatment [33], as well as to dual-agent ICI [34,35].
NRAS Mutation	Yes—NRAS-mutated tumors are associated with more aggressive phenotype [39,41,42].	No.
NF-1 Mutation	Yes—NF-1 mutations are associated with worse MSS [85].	Yes—NF-1 mutations confer resistance to BRAF/MEK-targeted treatment even in the setting of BRAF-mutated disease [86].
Nodular Subtype	Yes—Nodular subtype is associated with NRAS mutations, a more aggressive phenotype, and worse OS [52,53].	No.
Acral Subtype	Yes—AM is associated with worse OS compared to other subtypes of cutaneous melanoma [87].	Yes—Low TMB is associated with worse response to ICI [54,55].
PD-L1	No—Results are inconsistent.	No—Results are inconsistent.
ctDNA	Yes—Detectable ctDNA levels following treatment (surgery or systemic) confer worse OS [67,68,82].	Yes—Rising ctDNA levels during systemic therapy are associated with shorter DMFS in adjuvant setting and shorter PFS in the metastatic setting [88].
GEP	Yes—High-risk signatures are associated with a higher probability of SLN positivity [89] and worse MSS [77,78].	No.

## 4. Surgery for Metastatic Melanoma in the Modern Era

### 4.1. Contemporary Patient Selection

The role of surgery in metastatic melanoma has evolved markedly in the era of immune checkpoint inhibitors (ICIs). Prior to the introduction of ICIs in 2011, 5-year survival for patients with metastatic disease was less than 10%, with a median OS of only 6–7.5 months from the time of stage IV diagnosis [90,91]. The advent of immunotherapy and targeted therapy has dramatically improved long-term outcomes, with median OS reaching up to 72.1 months for patients treated with dual-ICI therapy in CheckMate 067 [92].

Despite these advances in systemic therapy, metastasectomy continues to play an important role in selected patients, particularly those with low-volume metastatic disease, oligoprogression, or minimal residual disease after therapy. Surgery can contribute to prolonged survival and symptom control, and it should remain an integral component of multidisciplinary discussions. A recent compelling example comes from Nelson et al., who evaluated over 2200 patients with stage IV melanoma and conducted a matched-pair analysis comparing surgical and non-surgical patients receiving contemporary systemic therapies. Among 47 matched pairs, surgery followed by modern therapy was associated with a significantly higher 5-year MSS compared with systemic therapy alone (58.8% vs. 38.9%, *p* = 0.049). It is also notable that the proportion of patients undergoing surgery in this analysis increased over the study period (54.5% vs. 44.7%, *p* = 0.02) [93].

Despite these findings, there remains no consensus defining which patients are optimal candidates for surgery. Therefore, decisions must be individualized within a multidisciplinary discussion, guided by patient performance status and underlying tumor biology. As discussed earlier in this review, selecting patients for metastatectomy remains guided by the clinical predictors of tumor biology. While several modern advances have emerged, traditional clinical variables continue to outweigh technologically derived biomarkers in current practice. Variables consistently associated with improved tumor biology include a longer disease-free interval, shorter tumor-doubling time, earlier stage of the primary tumor, involvement of fewer organs, and a lower number of metastatic lesions. These parameters remain highly relevant even in the era of advanced molecular profiling and modern systemic therapies. A recent large meta-analysis, including over 21,000 patients treated with contemporary systemic agents, demonstrated that patients selected for surgery had a significantly reduced risk of death compared with those receiving systemic therapy alone (hazard ratio 0.42; *p* < 0.0001) [94]. Patients with metastases confined to a single organ and those with a longer DFI experienced the most favorable outcomes.

Perhaps the most important newer clinical prognostic factor in the era of modern systemic therapy is the assessment of treatment response. This has emerged as a powerful predictor of favorable tumor biology in metastatic melanoma. A retrospective review of 128 patients by Medina et al. demonstrated that pre-operative response to ICI was the strongest predictor of improved OS following surgery. Patients who responded to ICI had a median OS of 84.3 months, compared with 42.9 months among non-responders [95]. These results have been further corroborated by a recent multicenter retrospective study, which reported a mean OS of 114 months in patients achieving at least a partial response prior to metastasectomy, compared with 60 months for those with stable or progressive disease [55].

These studies not only illustrate the value of using systemic response to inform treatment decisions, but they also reinforce the rationale for using systemic therapy as first-line treatment before surgery. The theoretical mechanism behind this sequence is two-fold. First, neoadjuvant systemic therapy has the potential to eradicate micrometastatic disease—small, undetectable clusters of cancer cells that may have already spread beyond the primary tumor site [46]. Secondly, front-line systemic therapy is thought to enhance therapeutic efficacy by priming the host’s antitumor immune response. Preclinical and clinical studies suggest that neoadjuvant therapy can increase tumor antigen release, promote dendritic-cell activation, and augment T-cell-mediated responses [96], and the heightened immunologic activation contributes to more durable disease control [97].

This concept formed the basis for SWOG S1801, a randomized phase II trial that compared two strategies in patients with resectable stage III–IV melanoma: neoadjuvant pembrolizumab followed by surgery and additional adjuvant therapy versus the traditional approach of surgery first with adjuvant pembrolizumab alone. Patients in the neoadjuvant pembrolizumab arm had significantly improved event-free survival (EFS)—72% at two years vs. 49% in the adjuvant-only arm [98]. Over half of resected tumors in the neoadjuvant arm exhibited a major pathologic response, underscoring the potential of pre-operative immunotherapy to induce deep tumor regression. The results of SWOG S1801 provide compelling evidence that neoadjuvant ICI should be strongly favored over a surgery first approach.

### 4.2. Surgery for Partial Responders

Surgical resection may benefit patients with metastatic melanoma who achieve a partial response to systemic therapy but have residual disease. In a retrospective analysis, 57% of treated stage IV patients—predominantly with M1c visceral metastases—underwent metastasectomy. Patients who responded to systemic therapy experienced improved survival, as median OS was 84.3 months for responders undergoing surgery versus 42.9 months for responders managed with systemic therapy alone. Surgery in patients who did not respond to systemic therapy did not experience a survival advantage, emphasizing the importance of treatment response as a critical selection criterion [95].

### 4.3. Surgery for Oligoprogressive Disease

Oligoprogression refers to a scenario in which limited progression occurs in a small subset of metastatic lesions, while the majority of disease remains controlled by systemic therapy. It reflects intratumoral heterogeneity, where a subset of resistant clones escapes the response to systemic therapy, and it affects approximately 5–10% of melanoma patients treated with ICI or targeted therapy [99,100]. When isolated treatment-resistant lesions are amenable to surgical clearance, resection of these progressors can be effective and appears to improve outcomes while allowing systemic therapy to maintain control of the responsive disease. This synergistic multimodal approach integrates the strengths of both the local (surgical) and systemic treatments [99,100].

Evidence supporting this was described by Bello et al. [101]. These authors reported on 237 advanced melanoma patients who underwent resection after ICI, and analyzed outcomes according to their response to therapy at the time of surgery. Group 1 patients had responding or stable disease (*n* = 12), and had an impressive 90% estimated 5-year survival. Group 2 were the ones with oligoprogression or one isolated progressing lesion (*n* = 106), and they had a 60% 5-year OS. Group 3 consisted of patients with multifocal progressive disease (*n* = 119), and they had a median OS of only 7.8 months and a 5-year OS of 6% (*p* < 0.0001).

These findings underscore the importance of treatment response as a predictive biomarker for favorable outcomes and selection for surgery. Notably, even among patients with oligoprogression (one isolated progressing lesion), long-term survival was achievable through surgery. Moreover, among oligoprogressers in Group 2, those who had complete resection of the progressing lesion (*n* = 71) had a 75% 5-year OS, while those with incomplete resection or remaining disease (*n* = 35) had only a 30% 5-year OS (*p* < 0.001) [101].

Similar findings were reported by Li et al., who analyzed 190 patients undergoing surgery after at least four weeks of ICI or targeted therapy [102]. Patients in whom all progressive lesions were resected had a 5-year OS exceeding 60%, even when residual responsive disease remained. Patients with residual progressive disease had a 5-year OS of only 8% [102]. These studies underscore the potential role of surgery in selectively managing resistant disease clones during systemic therapy, highlighting oligoprogression as a setting where metastasectomy can meaningfully extend survival.

**Figure 1:**
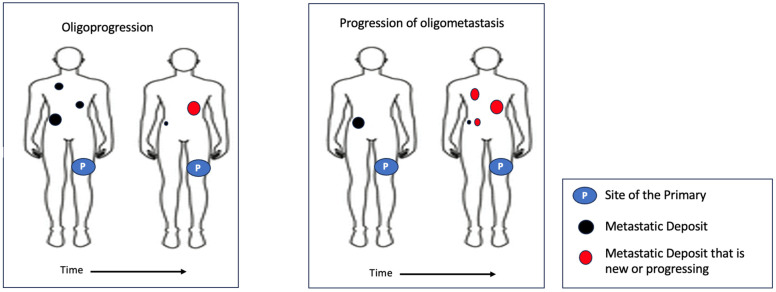
Animated explanation of oligoprogression versus progression of oligometastatic.

The concept of oligoprogression, defined as the development of a single isolated progressing lesion, should be distinguished from de novo oligometastasis that arises during treatment (Figure 1). Asher et al. described the latter concept, reporting that 29% of treatment-naive patients developed progressive disease while undergoing dual-agent ICI therapy, and experienced a median OS of 4.8 months [103]. These outcomes closely parallel those seen in Group 3 of the Bello et al. study [101], which involved multifocal progression rather than oligoprogression.

The role of surgery for oligoprogressive disease seems to also extend to patients who experience progression following adoptive cell transfer (ACT) as well. Klemen et al., from the Surgery Branch of the National Cancer Institute, reported outcomes of patients who underwent resection of progressive metastatic deposits after ACT [104]. Among 115 patients treated with ACT who initially achieved a response, but subsequently developed progressive metastatic disease, 23% underwent surgery. In this cohort, the median PFS was 11 months, and the 5-year OS was 57%.

### 4.4. Optimal Timing for Metastatectomy Following Systemic Treatment

When planning surgery in patients receiving ICIs, the timing of maximal treatment response is a critical consideration. However, response kinetics are highly variable. Clinical trials of ICIs in advanced melanoma demonstrate that many patients exhibit early tumor response. However, responses can continue to evolve over several months, and in some cases, years. In the phase Ib KEYNOTE 001 study of pembrolizumab for metastatic melanoma, the median time to initial response was 2.8 months, but the range extended from 0.5 to 49.6 months [58]. Moreover, some patients who initially achieve a partial response later convert to a complete response, with historical ipilimumab studies demonstrating a median time to complete response of approximately 30 months [105]. Ongoing responses have also been reported in patients who discontinue therapy early. In a phase I trial of nivolumab, over half of responding patients continued to respond for at least 4 months while off treatment [106].

Another decision-making challenge is distinguishing true tumor progression from pseudoprogression. Pseudoprogression is characterized by initial radiologic tumor enlargement due to immune cell infiltration rather than true tumor growth. Traditional response criteria such as RECIST may misclassify these patients as having progressive disease. To address this, immune-related response criteria (irRC) have been developed to account for transient increases in tumor size or new lesions before confirming progression [107,108]. These data emphasize the variability that can be observed in melanoma during ICI therapy. Moreover, it highlights the importance of allowing adequate time for maximal immunologic effect before considering additional interventions, such as surgery, in the multimodal management of metastatic melanoma.

### 4.5. Surgery for Palliation

In patients with metastatic melanoma where cure is no longer feasible, resection can occasionally offer a valuable palliation of debilitating symptoms such as pain, bleeding, obstruction, or neurologic compromise. In a seminal 1986 series of 65 patients who underwent palliative excision of 94 metastatic lesions, symptomatic relief was achieved in 77% of brain metastases, 100% of lung lesions, 88% of distant lymph/subcutaneous metastases, and 100% of abdominal metastases. Median OS ranged from 8 to 15 months depending on site, and 16% survived 2 years or longer [109]. Another report of emergency surgery for intra-abdominal complications (bleeding/obstruction) from melanoma metastases reported a median OS of 14 months, a 30-day mortality of 7%, and a 1-year OS of 64 % [110]. In a more recent series of patients receiving modern systemic therapies, patients undergoing small bowel resections for metastatic melanoma experienced an OS of 23.7 months for resections done for palliative intent [111]. On the other hand, Medina et al. noted in his series of resected melanoma metastasis that OS was not improved when palliation was the primary indication for resection [95]. These findings highlight that, in non-curative settings, surgical intervention can provide meaningful symptomatic relief and, in carefully selected patients, modest survival improvement.

## 5. Conclusions

Surgical resection continues to improve OS in selected patients with stage IV melanoma even in the era of modern systemic therapy. For most patients with metastatic melanoma, systemic therapy should serve as the first-line treatment, given its proven efficacy in controlling widespread disease. This principle should apply even to those patients with surgically resectable metastatic melanoma, as a neoadjuvant approach enables both host immune priming and evaluation of treatment response. Effective first-line therapy has extended surgical indications to include limited progressing lesions in oligoprogressive patients. Although decisions regarding surgical management still mandate a multidisciplinary assessment of clinical parameters, molecular profiling in addition to predictive biomarker assays, gene expression profiling, and circulating tumor DNA may provide additional insight into tumor biology, and they hold great promise to refine patient selection and personalize treatment in the future.

## Data Availability

No new data were created or analyzed in this study. Data sharing is not applicable to this article.
